# Microtopography mediates the climate–growth relationship and growth resilience to drought of *Pinus tabulaeformis* plantation in the hilly site

**DOI:** 10.3389/fpls.2022.1060011

**Published:** 2022-11-22

**Authors:** Hongming Zhao, Jiabing Wu, Anzhi Wang, Dexin Guan, Yage Liu

**Affiliations:** ^1^ Key Laboratory of Forest Ecology and Management, Institute of Applied Ecology, Chinese Academy of Sciences, Shenyang, China; ^2^ College of Resources and Environment, University of Chinese Academy of Sciences, Beijing, China

**Keywords:** tree ring, microtopography, *Pinus tabulaeformis*, plantation forests, drought response

## Abstract

Understanding the factors affecting the growth of plantation forests can reduce the loss of economic and ecological values caused by plantation forest subhealth. Plantation forests are widely distributed in hilly areas with microtopographic features. Microtopography influences climatic factors associated with plant growth, during not only general time but also extreme events like droughts. However, little research has been conducted on the effects of microtopography on the plantation forest growth. In this paper, we selected Pinus tabulaeformis planted in a hilly site, and studied the effect of microtopography on the climate–growth relationship and drought response of a typical plantation in Northeast China using dendroecological methods. We found: 1) Between hill positions, temperature caused a climatic growth difference. Compared to the hilltop, the correlation of annual growth on the hillside with monthly temperature was more negative in July–August and less positive in January–April. 2) Between aspects, precipitation intensities caused a climatic growth difference. Compared to the sunny slope, the correlation of annual growth on the shady slope with monthly total precipitation below 10 mm/day was less positive (May–June) or more negative (March–April and July), while that with monthly total precipitation above 10 mm/day was more positive in most months.3) Drought response varied significantly based on hill position and aspect. There was no significant difference in resistance between hill positions, while recovery and resilience on the hilltop were greater than those on the hillside.Resistance, recovery, and resilience were all lower on the sunny slope than those on the shady slope. Overall, microtopography exists the effects on the growth of plantation forests, both in terms of climate-growth relationships in general climate and in response to drought when extreme events. Meanwhile, the climatic factors that caused the difference in growth of plantation forests between hill positions and aspects differed. The difference in growth between hill positions was caused by temperature, while that between aspects was caused by precipitation intensity. Drought response difference reflected the legacy effect of drought on plantation growth, which could lead to subsequent changes in climate-growth relationships. These findings demonstrate that strengthening the research of forest trees on microtopography is necessary for accurate carbon sink assessment and precise forest management.

## Introduction

A plantation is an important component of terrestrial ecosystems and plays an increasingly important role in restoring and rejuvenating forest ecosystems, enhancing forest carbon sinks, and improving the ecological environment. China has the largest area of plantation forests in the world, and the area of plantation forests in Northeast China is enormous (Li et al., 2020). However, many of them are sub-healthy (Zhao et al., 2021). This could result in a huge loss of economic and ecological benefits. We need a better understanding of plantation growth.

Topography, especially mountain position and aspect, impacts radiation (Murphy et al., 2020; Zhirnova et al., 2020), temperature (Zhirnova et al., 2020; Wang and Yang, 2021), and soil moisture distribution and dynamics (Murphy et al., 2020; Zhirnova et al., 2020). These affect the climate–growth relationship of forests, which in turn affects forest biomass (Homeier et al., 2010; González-Jaramillo et al., 2018). Unfortunately, most current studies focus on large-scale topography with elevation differences of hundreds or even thousands of meters, such as the Changbai Mountains (Wang et al., 2013; Yu et al., 2013; Yu and Liu, 2020) and the Alps (Pappas et al., 2020), and there is a lack of research on small-scale topography. However, it has been demonstrated that these effects remain even on microtopography with elevation differences of less than 100 m (Galicia et al., 1999; Ma et al., 2020; O’Brien and Escudero, 2022). Furthermore, plantations spread widely in hilly areas with a small elevation difference. Therefore, research into the effect of microtopography on the climate–growth relationship of plantations is required.

The effect of microtopography on the distribution of climatic factors is also extant (Mei et al., 2018; Esteban et al., 2021), or even greater (Wang et al., 2020; O’Brien and Escudero, 2022), when precipitation decreases or even drought. The resilience index and its components, resistance and recovery indices, are commonly used to evaluate the effect of drought on plant growth. Resilience describes a tree’s ability to achieve growth rates similar to those before a drought. Defined in this way, resilience encompasses the ability to reduce the effect of the disturbance, i.e., resistance, and the ability to regain pre-disturbance levels of growth after drought, i.e., recovery (Lloret et al., 2011). At present, the drought response of trees is mostly researched on large-scale topography, such as the Iberian Range (Marques et al., 2016) and Helan Mountains (Wang et al., 2019; Zeng et al., 2020), and our understanding of it in hilly areas is not sufficient. Meanwhile, the effect of topography on the drought response of trees is not uniform in large-scale topography. For example, Bosela et al. (2020) found that the significance of resistance difference in different mountain positions is inconsistent across several sample plots, although they have similar elevation differences. Thus, even on a small scale, there is still uncertainty about whether trees on microtopography in hilly areas respond differently to drought.

*Pinus tabulaeformis* is an important plantation tree species in northern China. At present, its plantation area is 167.76 × 10^4^ ha, accounting for 2.9% of the total plantation area (National Forestry and Grassland Administration, 2019). In addition, *P. tabulaeformis* is a representative tree species in the low mountain and hilly areas, with a wide distribution on microtopography. Therefore, we used tree rings of *P. tabulaeformis* planted in the northern hill of Shenyang to investigate: 1) the effect of microtopography on the climate–growth relationship of a *P. tabulaeformis* plantation and 2) the effect of microtopography on growth resilience to a drought of a *P. tabulaeformis* plantation and whether it is significant. We expect the findings of this study will be useful for carbon sink assessment and forest management.

## Materials and methods

### Study site and climate data

**Figure 1:**
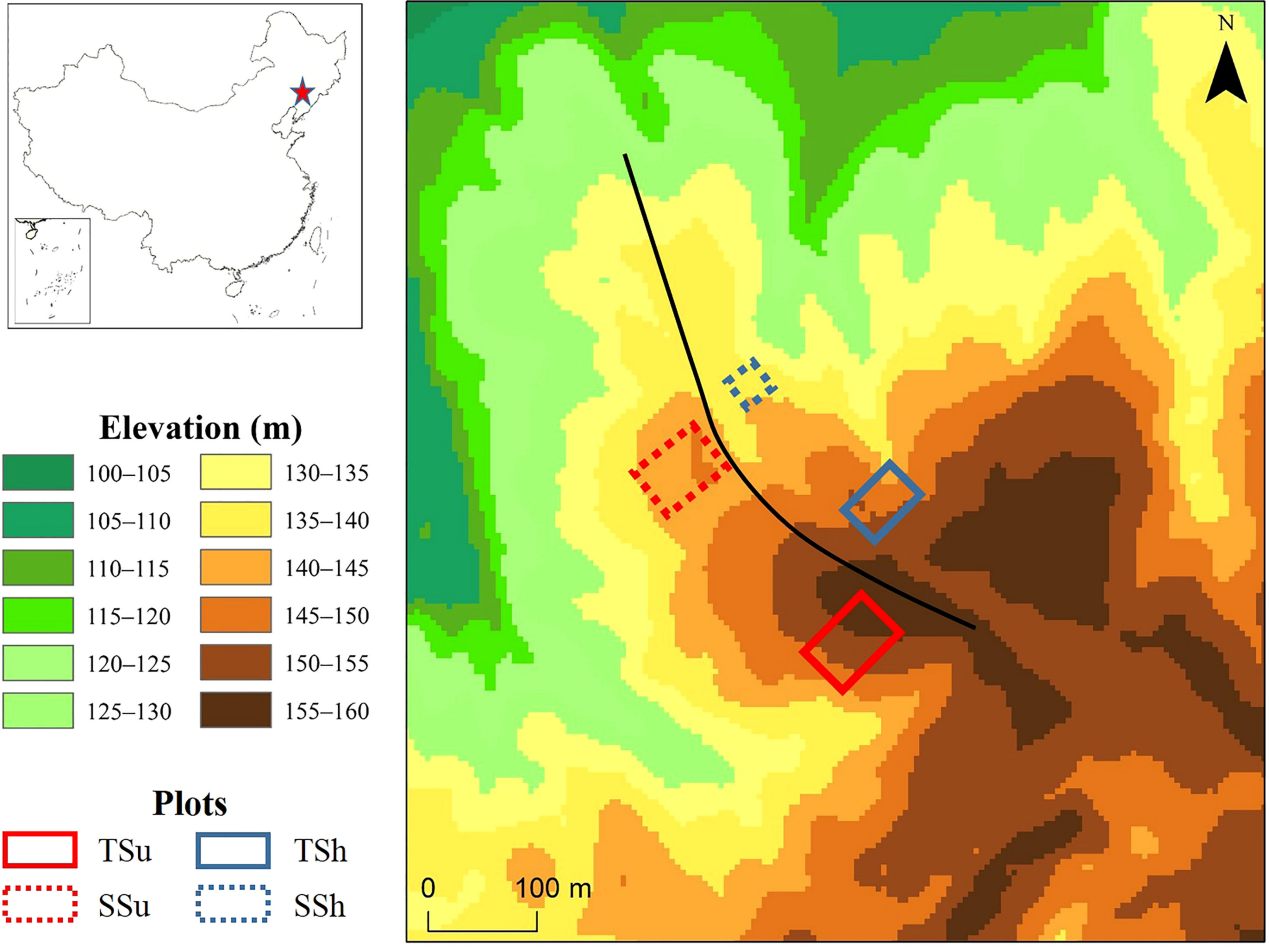
Site information and the topography of sample plots. The dashed squares indicate the hillside, and the solid squares indicate the hilltop. The red squares denote the sunny slope, and the blue squares denote the shady slope. The black curve represents the ridge line. The red star represents the study site.

The study site is in suburban Shenyang (41°54′N, 123°36′E), where the landform type is hilly and the soil is mainly brown soil and dark brown soil with a high gravel content. We established four plots with few traces of human activity on the sunny (the southwest side of the ridgeline) and shady (the northeast side of the ridgeline) slope of the hilltop (about 150 m a.s.l.) and the hillside (about 130 m a.s.l.) (**Figure 1**). They are abbreviated as TSu (the sunny slope of the hilltop), TSh (the shady slope of the hilltop), SSu (the sunny slope of the hillside), and SSh (the shady slope of the hillside). To obtain as many representative and valid samples as possible, we set the plot size as large as possible and ensured that the proportion of *P. tabulaeformis* in each plot was above 90%. The plot sizes (length × width) are 50 m × 75 m, 40 m × 60 m, 50 m × 70 m, and 30 m × 30 m, respectively. Among them, the area of the SSh is smaller than that of the other three, limited by the terrain scale and human activity. The average slope gradients of the plots are 20° (TSu), 16° (TSh), 19° (SSu), and 14° (SSh), respectively.

P. tabulaeformis dominated the study area, interspersed with Larix gmelinii, Ulmus pumila, and Quercus mongolica. The understory vegetation, consisting of Platycodon grandiflorus, Polygonatum humile, Rhamnus arguta, Menispermum dauricum, etc., had such little biomass that it could be viewed as negligible compared to the arbor. The average height of P. tabulaeformis was about 10 m, the diameter at breast height was mainly 15–25 cm, the crown density was about 0.7, and the age of the stand was about 50 years. On the sunny slope, the stand density was about 900 trees/ha, while on the shady slope, it was 500 trees/ha.

**Figure 2:**
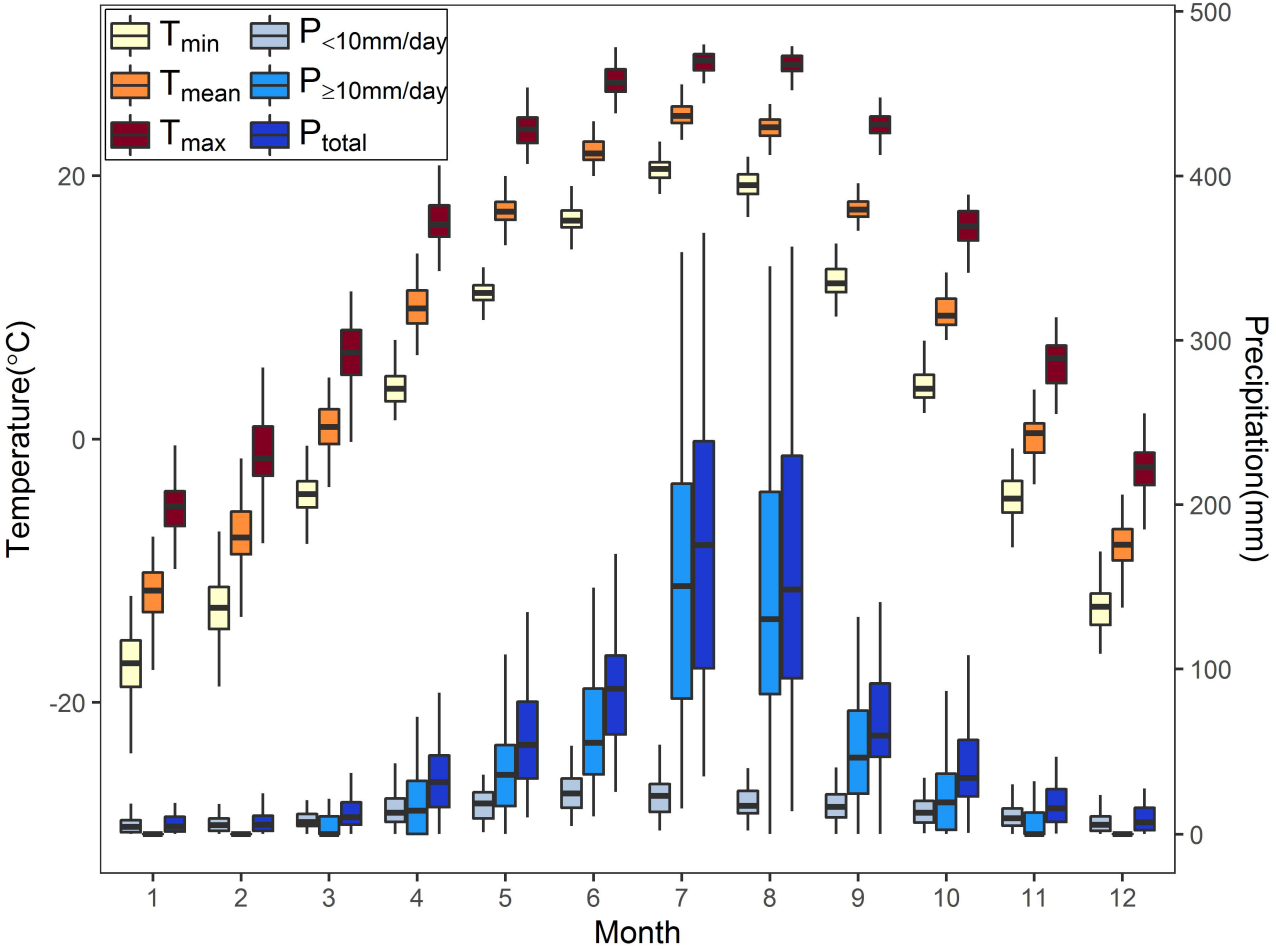
Monthly temperature and precipitation indices from January to December in the study area during 1951–2020. T_min_, T_mean_, and T_max_ represent the monthly averaged minimum, mean, and maximum temperature, respectively. P_<10mm/day_, P_≥10mm/day_, and P_total_ represent the monthly total precipitation below 10 mm/day, above or equal to 10 mm/day, and above 0 mm/day.

Climate data, including temperature and precipitation recordings of 1951–2020, come from the Shenyang meteorology station (downloaded from the China Meteorological Data Service Center, http://data.cma.cn), which is around 20 km away from the study site. The annual mean temperature was 8.21°C, and the annual total precipitation was 697.25 mm. The monthly mean, maximum, and minimum temperature (T_mean_, T_max_, T_min_) fluctuated relatively little and was at a high level in June–August (**Figure 2**). Precipitation was mainly concentrated in April–October, especially in July–August, when it was the highest throughout the year. The monthly precipitation above or equal to 10 mm/day (P_≥10mm/day_) accounted for a relatively high proportion in April–October, while the monthly precipitation below 10 mm/day (P_<10mm/day_) accounted for the majority in other periods (**Figure 2**).

### Soil sampling and measurement

In August 2020, we randomly selected nine points in each plot and used the excavation method to collect soil samples from 0–10-cm, 10–20-cm, and 40–50-cm depth soil layers. The soil and stones were separated by a 2-mm sieve, and the weight of each was recorded separately. Combined with the recorded volume of the sampling soil pit, we calculated the soil weight of 100 cm^3^ and filled it into the ring knife of the corresponding specification. The field capacity (FC) and saturated water content (SWC) of soil samples in each layer were measured using the ring knife method. In addition, the volumetric fraction of stones (VFS) was calculated as follows:


(1) $$\text{VFS}=V_{s}/V$$

(2) $$V_{s}=m_{s}/2.65$$

where *V_s_* was the volume of the stones, *V* was the volume of the sampling soil pit, *m_s_* was the weight of the stones, and 2.65 g/cm^3^ was the density of the stones (Liu, 1996).

### Tree ring sampling and chronology development

We selected some trees without insect pests as sample trees in each plot. Two cores per tree were extracted at chest height (about 1.3 m) from the orthogonal direction with an increment borer. Then, the samples were packaged and brought back to the laboratory. After fixing, drying, and grinding from coarse to fine until the tree rings were clearly visible, all sample cores were scanned and turned into pictures with an HP M277dw printer (1,200 dpi). We detected tree ring width (an accuracy of 0.01 mm) from the core pictures using the R package MtreeRing (Shi et al., 2019). The tree ring width data in each plot were checked and quality-controlled by the COFECHA program (Holmes, 1983). We detrended the tree ring data to remove the age-related signal using the negative exponential function and get the tree ring width index (TRWi). The TRWi series, grouped by plots and microtopography, were respectively averaged to build standard chronology (STD) using the robust Tukey bi-weight mean. Mean sensitivity, standard deviation, signal-to-noise ratio, first-order autoregressive coefficient, and expressed population signal (EPS) are the STD’s common statistical parameters (Fritts, 1972). Using 0.85 as the EPS threshold, the period above it for STD is considered reliable (Wigley et al., 1984). The STD statistics for all plots and microtopography are listed in **Tables 1** and **2**.

**Table 1:** Standard chronology (STD) statistics for all plots.

	TSu	TSh	SSu	SSh
Time span	1962–2020	1964–2020	1965–2020	1953–2020
Cores/Trees	98/54	30/16	79/47	28/15
Mean sensitivity	0.22	0.26	0.21	0.28
Standard deviation	0.28	0.30	0.32	0.33
Signal-to-noise ratio	29.42	14.52	24.99	6.12
First-order autoregressive coefficient	0.26	0.39	0.54	0.43
Expressed population signal (The start year of the EPS >0.85)	0.97 (1969)	0.94 (1971)	0.96 (1970)	0.86 (1980)

**Table 2:** Standard chronology (STD) statistics for all microtopography.

	Hilltop	Hillside	Sunny slope	Shady slope
Time span	1962–2020	1953–2020	1962–2020	1953–2020
Cores/Trees	128/70	107/62	177/101	58/31
Mean sensitivity	0.21	0.23	0.20	0.25
Standard deviation	0.28	0.31	0.29	0.30
Signal-to-noise ratio	33.55	25.37	39.94	15.67
First-order autoregressive coefficient	0.28	0.46	0.34	0.36
Expressed population signal (The start year of the EPS >0.85)	0.97 (1968)	0.96 (1970)	0.98 (1969)	0.94 (1971)

### Determination of drought events and computation of resilience indices

**Figure 3:**
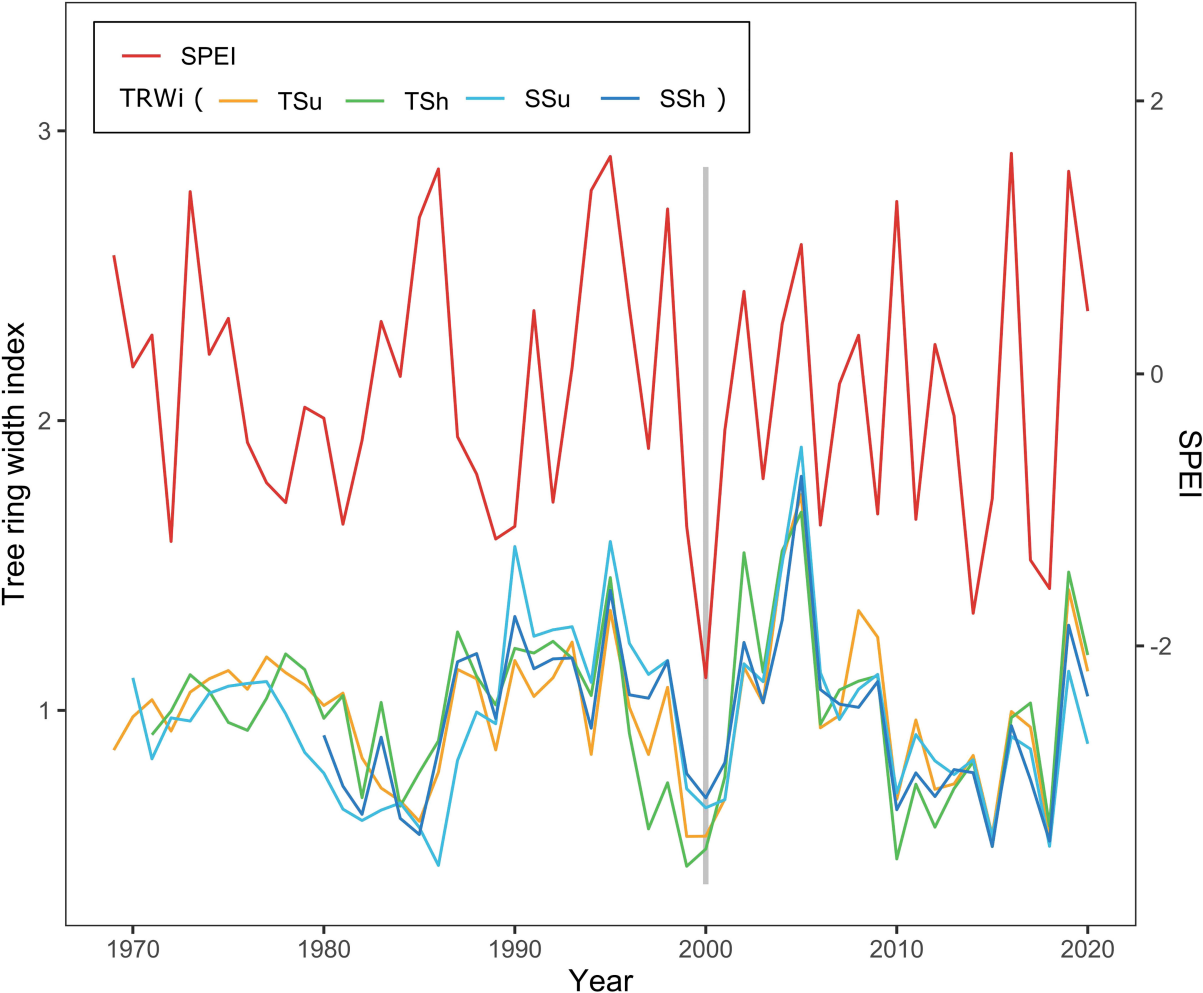
Standard chronologies (STDs) of the tree ring width index (TRWi) among all plots and Standardized Precipitation Evapotranspiration Index (SPEI) distribution show the drought event. The bar chart denotes the year of the drought event on all plots (2000).


In order to investigate the long-term radial growth response to drought, we analyzed the resilience and its components, resistance and recovery (Lloret et al., 2011). The drought intensity was detected using the Standardized Precipitation Evapotranspiration Index (SPEI) (Vicente-Serrano et al., 2010) during the key growing season (May–July). Referring to relevant research (DeSoto et al., 2020; Li et al., 2020), we considered the effects of the 4 years before and after the drought event. To find drought events associated with reduced growth, there were three criteria used (DeSoto et al., 2020): 1) the values of SPEI below the 10% percentile of SPEI distribution within the reliable period of the chronologies; 2) significantly low growth (over 5% reduction relative to the average growth of the previous 4 years) in the same year or the year after (considering the lag effect of climate–growth); and 3) in a 4-year time window, if there are multiple drought events that satisfy the previous two criteria, only the last one is taken as a drought event (to avoid growth recovery in previous drought events impacted by subsequent drought events). As shown in **Figure 3**, a drought event in all plots occurred in 2000. We computed the resilience indices to drought events as follows (Lloret et al., 2011; DeSoto et al., 2020):

(3) resistance=Dr/PreDr

(4) recovery=PostDr/Dr

(5) resilience=PostDr/PreDr=resistance×recovery

where Dr was defined as TRWi of the drought year, PreDr and PostDr represented separately mean TRWi of the preceding and post 4-year periods.

### Statistical analysis

**Figure 4:**
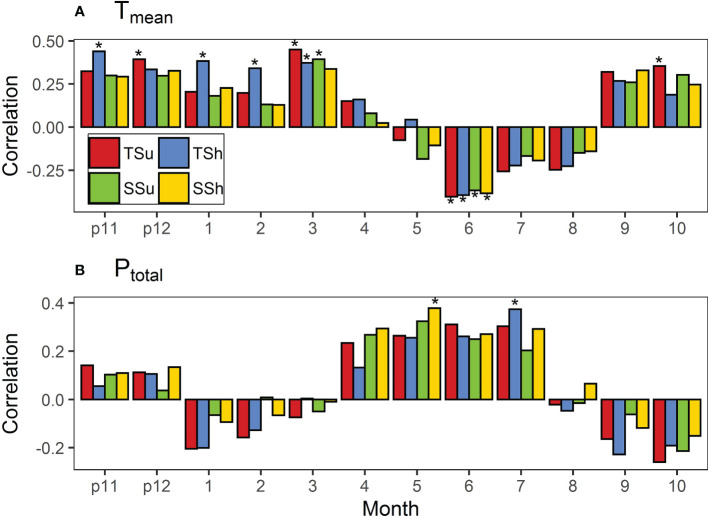
Correlations of standard chronologies (STDs) in all plots with **(A)** monthly mean temperature (T_mean_) and **(B)** total precipitation (P_total_). Bars marked with stars indicate that they reached the 0.05 significance level. “p11” and “p12” represent November and December of the previous year. Bars marked with * indicate that they reached the 0.05 significance level.

We chose the period from the previous November to the current October as a growing year of *P. tabulaeformis* (Cai et al., 2020). Since the roles of climate factors vary by month, we conducted a correlation analysis between the TRWi and the monthly temperature and precipitation indices for each month of the growing year. In order to eliminate the influence of variable autocorrelation, the first-order difference was used for the correlation analysis (Li et al., 2021). Each bar or color dot in the following correlation figures means the Pearson product-moment correlation coefficient between the first-order difference of STDs and that of monthly climatic factors. We chose the correlation between the first-order difference of STDs in all plots and that of the T_mean_ in March (**Figure 4A**) as a typical one to present in the form of a scatter plot (**Supplementary Figure S1**).

In addition to correlating the radial growth with climate variables (general effects), we also investigated the resilience indices (extreme effects) of the radial growth to drought events. The pairwise Wilcoxon rank sum test was used to detect whether the growth resilience to drought differed significantly among plots with different microtopographies.

To illustrate the effect of microtopography on radial growth, we combined and paired the plots by hill positions and aspects and performed the above analyses again. The Kruskal–Wallis rank sum test was used to detect whether the growth resilience to drought differed significantly between paired microtopography.

We used the R package SPEI (Santiago and Vicente-Serrano, 2017) to calculate the SPEI, and dplR (Bunn, 2008; Bunn, 2010) to detrend the raw tree ring width data and develop the chronologies. The rest of the operations and analyses were done in the R environment (R Core Team, 2021).

## Results

### Soil water storage capacity

In terms of soil water storage capacity, the shady slope was better than the sunny slope in all soil layers at the same hill position. Among the plots, except SSh, the deeper the soil layer, the greater the VFS, and the VFS in all layers of TSu were obviously the highest. The SWC and FC of SSh are slightly higher than those of SSu, and because of the VFS differences, the storage capacity of SSh will be further superior to that of SSu. Although the SWC of TSu was slightly higher than that of TSh, the water storage capacity of TSh would be better than that of TSu considering the significantly higher VFS in TSu (**Table 3**).

**Table 3:** Soil water storage capacity in all plots.

Depth (cm)	TSu			TSh			SSu			SSh		
	FC	SWC	VFS	FC	SWC	VFS	FC	SWC	VFS	FC	SWC	VFS
0–10	28.60%	53.40%	45.89%	30.86%	51.58%	6.43%	24.73%	42.29%	9.92%	27.04%	45.99%	9.16%
1–10	29.81%	50.23%	55.39%	29.07%	49.07%	11.02%	21.26%	36.59%	19.45%	28.82%	44.48%	8.48%
20–50	28.70%	46.64%	60.35%	23.97%	42.49%	17.58%	22.30%	39.76%	21.29%	29.88%	45.16%	7.91%

FC, field capacity; SWC, saturation water content; VFS, volumetric fraction of the stones. The units of FC, SWC, and VFS are %vol.

### Climate–growth relationships and resilience to drought among plots

The T_mean_ for multiple months was significantly correlated with TRWi in plots, but the direction shifted over time (**Figure 4** **)**. It was found that the T_mean_ in each month of May–August basically had a negative effect on the radial growth among all plots while having a positive effect in the other months. The T_mean_ in June had a significant negative effect on radial growth in all plots, while the T_mean_ in the previous November, March, and October had a significant positive effect on the radial growth in a few plots. Moreover, the T_mean_ has no significant effect on radial growth in other periods among all plots.

The correlations between STDs and the P_total_ in plots were not as significant as those between STDs and the T_mean_, but again the direction varied across time (**Figure 4**). STD correlations with the P_total_ were positive in all plots during the previous November–December and April–July months while negative in the other months. The P_total_ only had a significant effect on the radial growth in a few plots in May and July.

**Figure 5:**
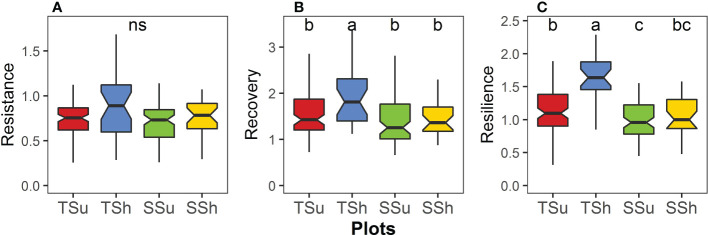
Differences in **(A)** resistance, **(B)** recovery, and **(C)** resilience among plots. We compare significance by pairwise Wilcoxon rank sum test (P < 0.05). “ns” means no significant difference.

Among the plots, the resistance had no significant difference, the recovery had certain significant differences, and the resilience had the most significant differences (**Figure 5**). The recovery on TSh was significantly higher than that on the other three plots, and there was no significant difference among the other three plots (**Figure 5B**). The resilience on the TSh was significantly higher and was significantly lower on the SSu than on the other plots, except SSh (**Figure 5C**). The difference in resistance, recovery, and resilience between aspects on the hilltop is larger than that on the hillside, and the difference in resistance, recovery, and resilience between hill positions on the shady slope is larger than that on the sunny slope (**Figure 5**).

### Contrasts between hill positions in climate–growth relationships and resilience to drought

**Figure 6:**
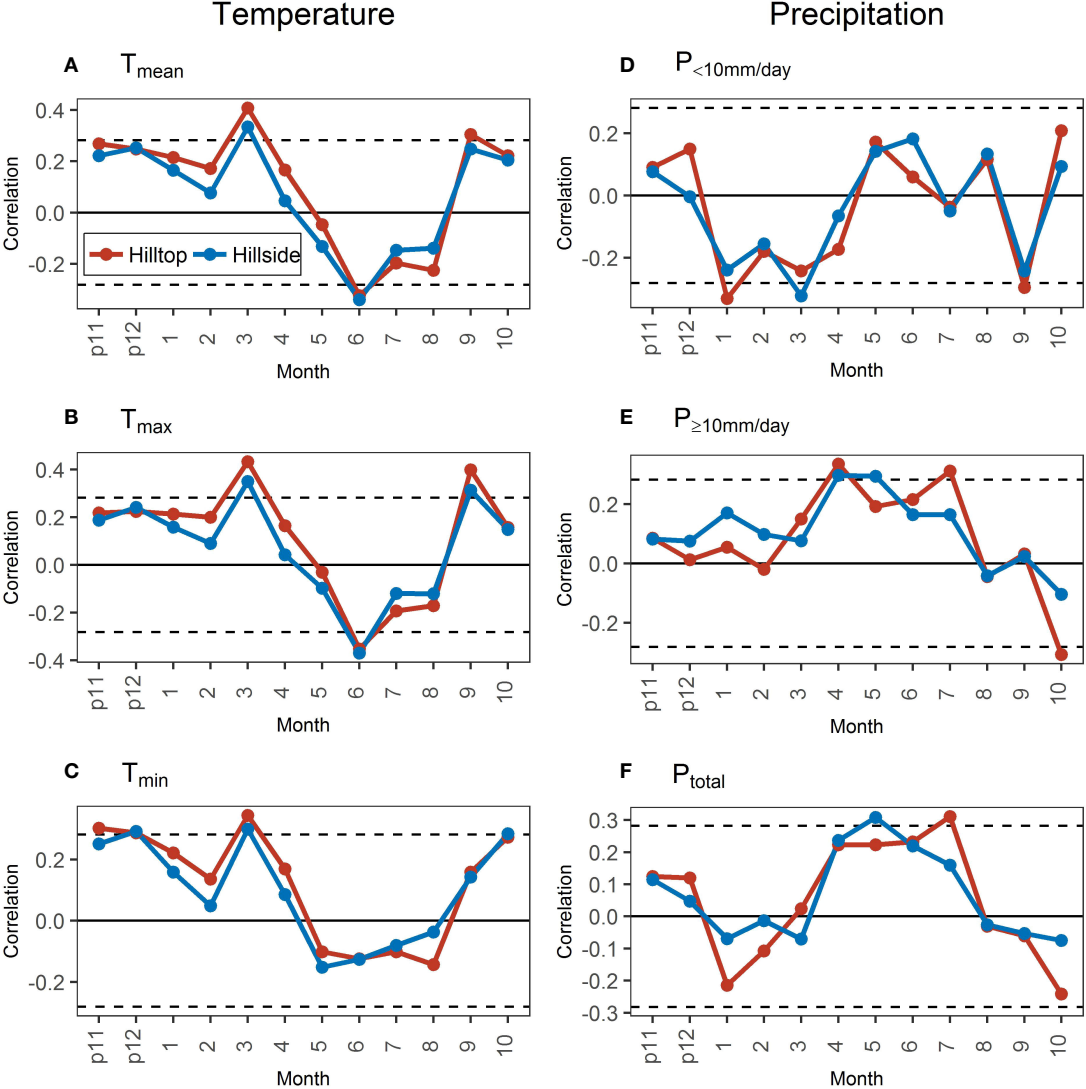
Correlations of standard chronologies (STDs) with monthly temperature **(A–C)** and precipitation **(D–F)** variables in the hilltop and the hillside during 1971–2020. The dotted black lines represent the significance level at 0.05. “p11” and “p12” represent November and December of the previous year.

The correlations of STDs with temperature variables showed obvious differences in January–April and July–August between hill positions (**Figures 6A-C**). The temperature variables were more positive for the growth on the hilltop than that on the hillside during the non-growing and early growing season (January–April, low evaporation) and more negative during the mid-growing season (July–August, high evaporation). These differences were generally observed for all temperature variables. However, the correlation of STDs with different temperature variables still had some differences, especially with the T_min_. The correlations of STDs with the T_mean_ and T_max_ in June have reached a significant level both on the hilltop and the hillside, but there was no significant correlation with the T_min_. Meanwhile, the correlation of STDs with the T_min_ in October was significant and greater than that in September, whereas the correlation with the T_mean_ and T_max_ nearly or actually reached significance in September and greater than that in October.

Between hill positions, the correlations of STDs with different intensities of precipitation were not as highly similar as they were with different temperature indicators (**Figures 6D-F**). Meanwhile, the temporal dynamics of the correlation difference in STDs with precipitation revealed little. Only in the previous November–February, the correlation of STDs with the P_≥10mm/day_ on the hillside was consistently more positive than that on the hilltop (**Figure 6E**).

**Figure 7:**
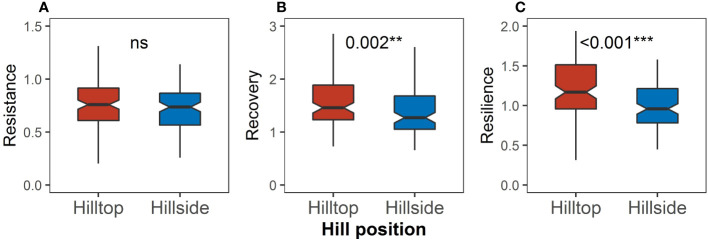
Differences in **(A)** resistance, **(B)** recovery, and **(C)** resilience between the hilltop and the hillside. Asterisks indicate significant differences between the hilltop and the hillside (Kruskal–Wallis rank sum test: **P < 0.01; ***P < 0.001). “ns” means no significant difference.

There was no significant difference in resistance between the hilltop and on the hillside (**Figure 7A**), but the recovery on the hilltop was significantly higher than that on the hillside (P = 0.002, **Figure 7B**), and the resilience on the hilltop was significantly higher than that on the hillside (P < 0.001, **Figure 7C**).

### Contrasts between aspects in climate–growth relationships and resilience to drought

**Figure 8:**
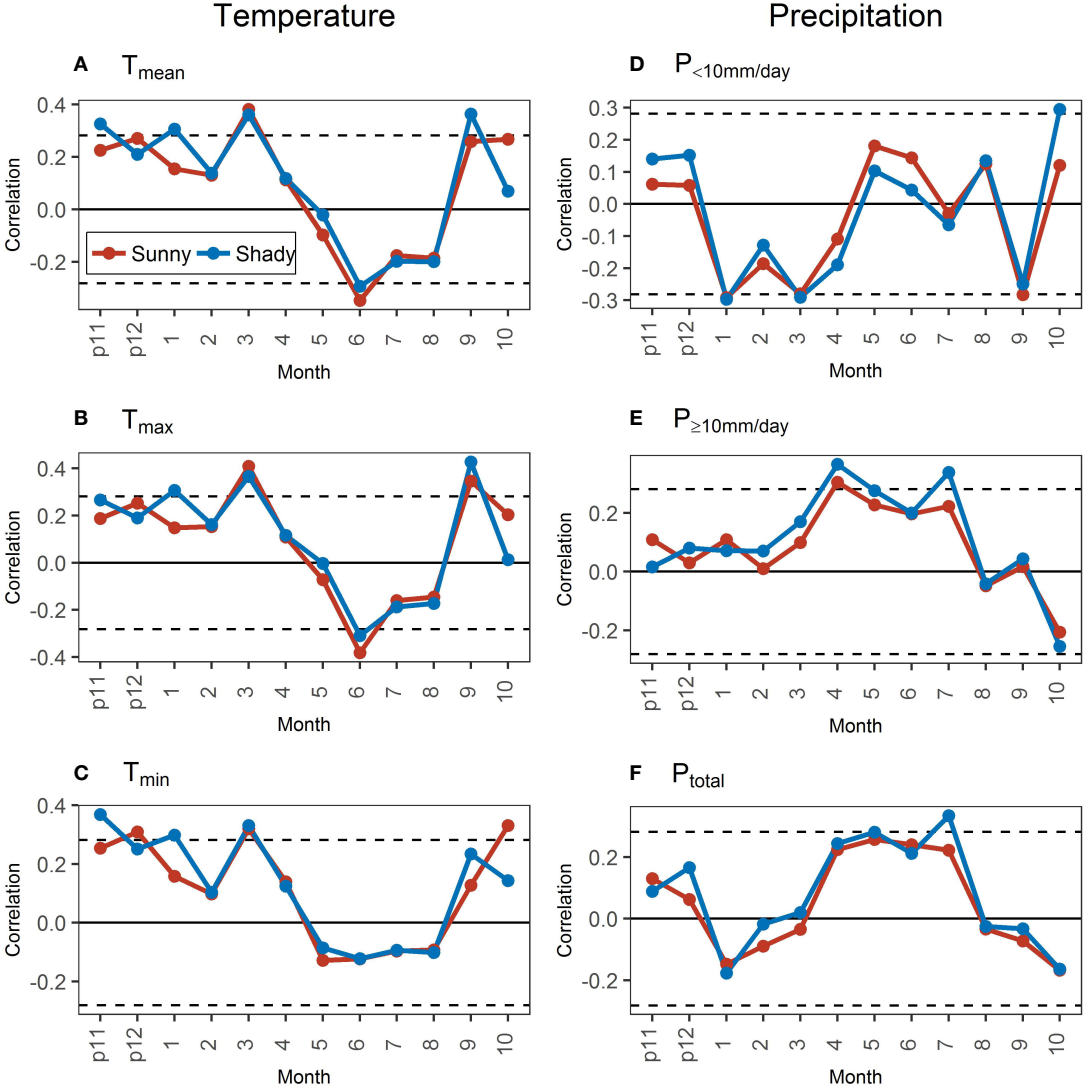
Correlations of standard chronologies (STDs) with monthly temperature **(A–C)** and precipitation **(D–F)** variables in the sunny and shady slopes during 1971–2020. The dotted black lines represent the significance level at 0.05. “p11” and “p12” represent November and December of the previous year.

The correlations of STDs and temperature variables between aspects showed a small difference in most months (**Figures 8A-C**), contrary to the larger differences between hill positions (**Figures 6A-C**). However, the particularity of T_min_ still existed between aspects. Specifically, the correlation was not significant in June and not significant in September along with less than that in October (**Figure 8C**).

The differences in the correlation of STDs with precipitation variables between aspects had clear temporal dynamics, contrary to the indistinct temporal dynamics between hill positions. Specifically, the correlations of STDs with precipitation variables all showed stable and persistent differences in March–July between hill positions, which were not shown between aspects. However, the correlation characteristics with different precipitation intensities were still clearly different (**Figures 8D-F**). In the previous November–March, when the precipitation was mainly <10 mm/day (**Figure 2**), the correlation of STD with P_<10mm/day_ in the sunny slope was less positive (previous November–December) or more negative (February) than or close to that in the shady slope. For the correlation with P_≥10mm/day_ in this period, the difference was small between aspects, with no obvious temporal dynamics. In April–October, the precipitation was mainly ≥10 mm/day (**Figure 2**). However, the differences in the precipitation–growth relationship between aspects in April–July differed from that in August–October. In April–July, the correlation of growth with P_<10mm/day_ in the shady slope was less positive (May–June) or more negative (April and July) than that in the sunny slope, and the correlation of growth with P_≥10mm/ day_ in the shady slope was more positive than that in the sunny slope. In August–October, the correlations of growth with precipitation between different aspects were similar, except that the correlation with P_<10mm/day_ in October was significantly higher in the sunny slope than that in the shady slope. The correlation of growth with the P_total_ in each month was similar to the correlation with the precipitation dominating in the corresponding month, the same as that in hill positions (**Figures 8D-F**).

**Figure 9:**
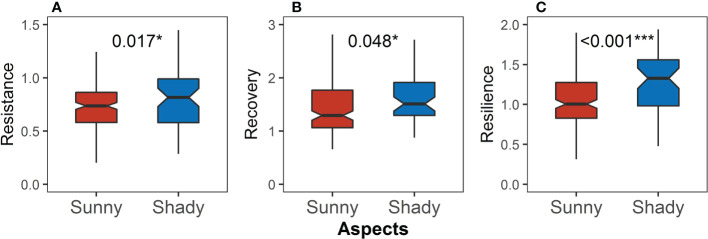
Differences in **(A)** resistance, **(B)** recovery, and **(C)** resilience between the sunny and shady slopes. Asterisks indicate significant differences between the sunny and shady slopes (Kruskal–Wallis rank sum test: *P < 0.05; ***P < 0.001).

Resistance, recovery, and resilience on the shady slope all were significantly higher than those on the sunny slope (P = 0.017, **Figure 9A**; P = 0.048, **Figure 9B**; P <0.001, **Figure 9C**), and the difference in resilience was more pronounced.

## Discussion

### Character distinctions of climate–growth relationships between paired microtopography

During the mid-growing season (July–August, high evaporation), the hillside showed a more positive correlation between growth and temperature than the hilltop. This is contrary to the findings of other studies, where the correlation of growth with the temperature at low elevation was more negative than at high elevation during the mid-growing season (Wang et al., 2015; Huo et al., 2017; Fernandez-de-Una et al., 2018). The main reason can be that the elevation difference of large mountains is large, and the temperature difference between different mountain positions is mainly related to the cooling caused by the elevation rise. However, in this study, the elevation difference between hill positions is less than 50 m, and the reason for the temperature difference is different from the former. In the hilly areas, the scale of the hill is smaller and more susceptible to the influence of the surrounding hills. Lower elevations are more likely to receive less radiation due to the shading of the surrounding hills. Thus, the temperature on the hillside is lower than that on the hilltop, which is the opposite of the phenomenon observed in large mountains. At the same time, growth on the hilltop was more sensitive to temperature because of less shade from the surrounding hills. Specifically, radiation variation was large on the hilltop due to less shade from the surrounding hills. The subsequent large temperature variation accentuated the effect of temperature. As a result, the growth on the hilltop benefited more from the positive effect of temperature during the non-growing and early growing season (January–April, low evaporation), and the correlation with temperature was more positive than that on the hillside.

The correlations of STDs with temperature variables between aspects were not obviously different in most months. We speculated that this was due to the small spatial scale of the hill and gentle gradient (average gradient is all around 17°), which limited the redistribution effect of aspect on radiation. The redistribution effect of topography on radiation is related to its spatial scale (Zhou and Chen, 2018). The spatial scale of the hills is much smaller than that of large-scale terrains such as mountains and plateaus, and the height difference of our study site is less than 50 m, so the aspect has a limited effect on the distribution of radiation.

During the main growing season (March–July), the correlation of STDs with different intensities of precipitation differed clearly between aspects, and the precipitation intensity that can benefit growth more (or suffer less) was different between aspects (**Figure 8**). During the same period, the positive effect of P_<10mm/day_ on the growth on the shady slope was weaker (less negatively during March–April and July), while the positive effect of P_≥10mm/day_ on it was stronger. This was not only due to the difference in evaporation caused by the redistribution of radiation through the aspect but also due to the difference in the utilization of precipitation at different intensities caused by a slightly steeper sunny slope than a shady slope. The greater the slope gradient, the lower the utilization of heavy precipitation (Singh et al., 2013; Mei et al., 2018). As well, the water storage capacity of the shady slope was greater than that of the sunny slope (**Table 3**), allowing the shady slope to better utilize the high-intensity precipitation. This further made the correlation of growth with P_≥10mm/day_ in the shady slope greater than that in the sunny slope. Whereas, when the precipitation intensity was low, the redistribution effect of the slope on precipitation and the water storage capacity advantage of the shady slope was reduced, then the shady slope with a lower water demand showed growth that was less positively (or more negatively) correlated with P_<10mm/day_.

### Character distinctions of growth resilience to drought between paired microtopography

Resistance on the shady slope was significantly higher (**Figure 9A**). This could be attributed to the difference in moisture situation between aspects. The wetter the area, the higher the resistance (Choat et al., 2018; Gao et al., 2021; Hong et al., 2021). When precipitation is less, the soil moisture difference between aspects will become larger (Wang et al., 2020). There is less precipitation during drought, and less water loss helps avoid worse water conditions. The aspect significantly regulates solar radiation, affecting land surface temperature and potential evapotranspiration (Mei et al., 2018; Zhirnova et al., 2020). Benefiting from this, the shady slope had more moisture during drought, making the resistance on it higher.

Recovery was significantly lower on the hillside and sunny slope (**Figures 7B**, **9B**). This could be related to the difference in radiation between hill positions and moisture situation between aspects. Recovery was highly connected with the photosynthetic capacity of plants (Galiano et al., 2011; Peltier and Ogle, 2020). Relatively little radiation made it difficult for the hillside (easier to be shaded by the surrounding hills) to produce enough non-structural carbohydrates to supplement the consumption during drought and support the radial growth after drought, resulting in lower recovery (**Figure 7B**). Moreover, water was an indispensable component of the photosynthesis process. Precipitation returned to normal levels in non-drought years, and the shady slope had advantages in photosynthesis due to better water storage, so the recovery was higher (**Figure 9B**).

Resilience was closely related to resistance and recovery. Although there was no significant difference in resistance between hill positions, significantly higher recovery resulted in significantly higher resilience on the hilltop (**Figure 7**). Because of significantly higher resistance and recovery, the resilience on the shady slope was significantly higher than that on the sunny slope (**Figure 9**).

Interestingly, the difference in resilience or its components (resistance and recovery) due to a 30-m elevation difference in this paper was similar to that due to a 120–800-m elevation difference in other studies (Wang et al., 2019; Bosela et al., 2020; Dell’Oro et al., 2020; Du et al., 2022). This means that there may be significant drought response differences even in the small-scale terrain, and the amount of difference in resilience-related indicators (recovery, resistance, or resilience) cannot be estimated only by the elevation difference. Other factors may also affect the response of tree growth to drought, thereby causing differences in resilience-related indicators of forests, such as the severity of drought in drought events (Wang et al., 2019; Du et al., 2022), altitude range of forests (Bose et al., 2020; Bosela et al., 2020), tree species (Li et al., 2020), and tree age (Wang et al., 2019).

### Potential response of growth on microtopography to climate change

With advancing climate change, the climatic factors causing the growth difference between hill positions and aspects will also change, such as temperature (climate warming) and precipitation intensity (an increase in heavy precipitation events) (Thc et al., 2015). Since there was no hill position that consistently benefited more (or suffered less) from increased temperature and heavy precipitation events throughout the growing season (**Figure 6**), the effect of climate change on growth differences between hill positions cannot be assessed through the climate–growth relationship. However, radial growth on the shady slope would benefit more (or suffer less) from these factor variations induced by climate change (**Figure 8**), leading to differences in growth and, even more so, drought responses increasing across aspects.

We observed significantly greater recovery and resilience of growth on the hilltop (**Figure 7**) and the shady slope (**Figure 9**). Recovery and resilience reflect a comprehensive and intrinsic ability of trees to regain predrought growth status after drought (Lloret et al., 2011). These findings indicated that the legacy effect of drought on different microtopographies occurred and differed, which may cause changes in the subsequent climate–growth relationship (Peltier and Ogle, 2020), and even increased the risk of death (Trugman et al., 2018; DeSoto et al., 2020). Considering that drought events occurred more frequently under climate change (Thc et al., 2015), from the perspective of drought response, the radial growth on the hilltop and the shady slope will be less adversely affected by climate change.

## Conclusions

In this study, we explored the climate–radial growth relationship and growth resilience to a drought of *P. tabulaeformis* plantations on microtopography, including different hill positions and aspects. It was found that there were still differences in the climate–radial growth relationship and significant drought response contrasts between hill positions and aspects on the hilly areas at small scales. Moreover, the main climatic factors that caused the difference in growth differed between different paired microtopographies. Temperature caused the difference in growth between hill positions, while it was precipitation intensity between aspects. Therefore, we believe that enhanced research on forest growth on microtopography will contribute to more accurate carbon sink assessment and better forest management. Additionally, the difference in drought indicated the legacy effect of drought on plantation growth, which could lead to consequent changes in climate-growth relationships.

## Data availability statement

The raw data supporting the conclusions of this article will be made available by the authors, without undue reservation.

## Author contributions

HZ and DG designed the study. HZ analyzed the data and visualized the data with the help of YL. HZ wrote the first version of the manuscript. JW, AW, and DG revised the manuscript. All authors contributed to the article and approved the submitted version.

## Funding

This work was supported by the National Key R&D Program of China (2019YFF0303203) and the National Natural Science Foundation of China (32171873, 31971728).

## Acknowledgments

We thank Chunjuan Gong, Haoyu Diao, Yuan Zhang, Jinyuan Tian, Chen Cui, and Junhui Yang for their support with the investigation.

## Conflict of interest

The authors declare that the research was conducted in the absence of any commercial or financial relationships that could be construed as a potential conflict of interest.

## Publisher’s note

All claims expressed in this article are solely those of the authors and do not necessarily represent those of their affiliated organizations, or those of the publisher, the editors and the reviewers. Any product that may be evaluated in this article, or claim that may be made by its manufacturer, is not guaranteed or endorsed by the publisher.

## References

[B1] Bose A. K. Gessler A. Bolte A. Bottero A. Buras A. Cailleret M. . (2020). Growth and resilience responses of scots pine to extreme droughts across Europe depend on predrought growth conditions. Glob. Change Biol. 26, 4521–4537. doi: 10.1111/gcb.15153 PMC7383776 32388882

[B2] Bosela M. Tumajer J. Cienciala E. Dobor L. Kulla L. Marcis P. . (2020). Climate warming induced synchronous growth decline in Norway spruce populations across biogeographical gradients since 2000. Sci. Total Environ. 752, 141794. doi: 10.1016/j.scitotenv.2020.141794 32898800

[B3] Bunn A. G. (2008). A dendrochronology program library in r (dplR). Dendrochronologia 26, 115–124. doi: 10.1016/j.dendro.2008.01.002

[B4] Bunn A. G. (2010). Statistical and visual crossdating in r using the dplR library. Dendrochronologia 28, 251–258. doi: 10.1016/j.dendro.2009.12.001

[B5] Cai L. X. Li J. X. Bai X. P. Jin Y. T. Chen Z. J. (2020). Variations in the growth response of pinus tabulaeformis to a warming climate at the northern limits of its natural range. Trees-Struct. Funct. 34, 707–719. doi: 10.1007/s00468-019-01950-2

[B6] Choat B. Brodribb T. J. Brodersen C. R. Duursma R. A. Lopez R. Medlyn B. E. (2018). Triggers of tree mortality under drought. Nature 558, 531–539. doi: 10.1038/s41586-018-0240-x 29950621

[B7] Dell’Oro M. Mataruga M. Sass-Klaassen U. Fonti P. (2020). Climate change threatens on endangered relict Serbian spruce. Dendrochronologia 59, 125651. doi: 10.1016/j.dendro.2019.125651

[B8] DeSoto L. Cailleret M. Sterck F. Jansen S. Kramer K. Robert E. M. R. . (2020). Low growth resilience to drought is related to future mortality risk in trees. Nat. Commun. 11, 545. doi: 10.1038/s41467-020-14300-5 31992718 PMC6987235

[B9] Du D. Jiao L. Chen K. Liu X. Qi C. Xue R. . (2022). Response stability of radial growth of Chinese pine to climate change at different altitudes on the southern edge of the tengger desert. Glob. Ecol. Conserv. 35, e02091. doi: 10.1016/j.gecco.2022.e02091

[B10] Esteban E. J. L. Castilho C. V. Melgaço K. L. Costa F. R. C. (2021). The other side of droughts: wet extremes and topography as buffers of negative drought effects in an Amazonian forest. New Phytol. 229, 1995–2006. doi: 10.1111/nph.17005 33048346

[B11] Fernandez-de-Una L. Aranda I. Rossi S. Fonti P. Canellas I. Gea-Izquierdo G. (2018). Divergent phenological and leaf gas exchange strategies of two competing tree species drive contrasting responses to drought at their altitudinal boundary. Tree Physiol. 38, 1152–1165. doi: 10.1093/treephys/tpy041 29718459

[B12] Fritts H. C. (1972). Tree rings and climate. Sci. Am. 226, 92–100. doi: 10.1038/scientificamerican0572-92

[B13] Galiano L. Martinez-Vilalta J. Lloret F. (2011). Carbon reserves and canopy defoliation determine the recovery of scots pine 4 yr after a drought episode. New Phytol. 190, 750–759. doi: 10.1111/j.1469-8137.2010.03628.x 21261625

[B14] Galicia L. López-Blanco J. Zarco-Arista A. E. Filips V. Garcıía-Oliva F. (1999). The relationship between solar radiation interception and soil water content in a tropical deciduous forest in Mexico. Catena 36, 153–164. doi: 10.1016/S0341-8162(98)00121-0

[B15] Gao J. Yang B. Peng X. Rossi S. (2021). Tracheid development under a drought event producing intra-annual density fluctuations in the semi-arid China. Agric. For. Meteorol. 308–309, 108572. doi: 10.1016/j.agrformet.2021.108572

[B16] González-Jaramillo V. Fries A. Zeilinger J. Homeier J. Paladines-Benitez J. Bendix J. (2018). Estimation of above ground biomass in a tropical mountain forest in southern Ecuador using airborne LiDAR data. Remote Sens. 10, 660. doi: 10.3390/rs10050660

[B17] Holmes R. L. (1983). Computer-assisted quality control in tree-ring dating and measurement. Tree-Ring Bull. 43, 69–78.

[B18] Homeier J. Breckle S.-W. Günter S. Rollenbeck R. T. Leuschner C. (2010). Tree diversity, forest structure and productivity along altitudinal and topographical gradients in a species-rich Ecuadorian montane rain forest. Biotropica 42, 140–148. doi: 10.1111/j.1744-7429.2009.00547.x

[B19] Hong Y. Zhang L. Liu X. Aritsara A. N. A. Zeng X. Xing X. . (2021). Tree ring anatomy indices of pinus tabuliformis revealed the shifted dominant climate factor influencing potential hydraulic function in western qinling mountains. Dendrochronologia 70, 125881. doi: 10.1016/j.dendro.2021.125881

[B20] Huo Y. X. Gou X. H. Liu W. H. Li J. B. Zhang F. Fang K. Y. (2017). Climate-growth relationships of schrenk spruce (Picea schrenkiana) along an altitudinal gradient in the western tianshan mountains, northwest China. Trees-Struct. Funct. 31, 429–439. doi: 10.1007/s00468-017-1524-8

[B21] Li M.-Y. Fang L.-D. Duan C.-Y. Cao Y. Yin H. Ning Q.-R. . (2020). Greater risk of hydraulic failure due to increased drought threatens pine plantations in horqin sandy land of northern China. For. Ecol. Manage. 461, 117980. doi: 10.1016/j.foreco.2020.117980

[B22] Li J. X. Song F. B. Jin Y. T. Yun R. X. Chen Z. J. Lyu Z. Y. . (2021). Critical temperatures controlling the phenology and radial growth of pinus sylvestris var. mongolica on the southern margin of a cold temperate coniferous forest. Ecol. Indic. 126, 107674. doi: 10.1016/j.ecolind.2021.107674

[B23] Liu G. (1996). Soil physical and chemical analysis and description of soil profiles (Beijing, China: Chinese Standard Press).

[B24] Lloret F. Keeling E. G. Sala A. (2011). Components of tree resilience: effects of successive low-growth episodes in old ponderosa pine forests. Oikos 120, 1909–1920. doi: 10.1111/j.1600-0706.2011.19372.x

[B25] Marques L. Julio Camarero J. Gazol A. Zavala M. A. (2016). Drought impacts on tree growth of two pine species along an altitudinal gradient and their use as early-warning signals of potential shifts in tree species distributions. For. Ecol. Manage. 381, 157–167. doi: 10.1016/j.foreco.2016.09.021

[B26] Ma H. Zhu Q. Zhao W. (2020). Soil water response to precipitation in different micro-topographies on the semi-arid loess plateau, China. J. For. Res. 31, 245–256. doi: 10.1007/s11676-018-0853-9

[B27] Mei X. Zhu Q. Ma L. Zhang D. Liu H. Xue M. (2018). The spatial variability of soil water storage and its controlling factors during dry and wet periods on loess hillslopes. Catena 162, 333–344. doi: 10.1016/j.catena.2017.10.029

[B28] Murphy P. C. Knowles J. F. Moore D. J. P. Anchukaitis K. Potts D. L. Barron-Gafford G. A. (2020). Topography influences species-specific patterns of seasonal primary productivity in a semiarid montane forest. Tree Physiol. 40, 1343–1354. doi: 10.1093/treephys/tpaa083 32597974

[B29] National Forestry and Grassland Administration (2019). China Forest resources report 2014-2018) (Beijing, China: China Forestry Press).

[B30] O’Brien M. J. Escudero A. (2022). Topography in tropical forests enhances growth and survival differences within and among species *via* water availability and biotic interactions. Funct. Ecol. 36, 686–698. doi: 10.1111/1365-2435.13977

[B31] Pappas C. Peters R. L. Fonti P. (2020). Linking variability of tree water use and growth with species resilience to environmental changes. Ecography 43, 1386–1399. doi: 10.1111/ecog.04968

[B32] Peltier D. M. P. Ogle K. (2020). Tree growth sensitivity to climate is temporally variable. Ecol. Lett. 23, 1561–1572. doi: 10.1111/ele.13575 33463045

[B33] R Core Team (2021). R: A language and environment for statistical computing (Vienna, Austria: R Foundation for Statistical Computing). Available at: https://www.R-project.org/.

[B34] Santiago B. Vicente-Serrano S. M. (2017). SPEI: Calculation of the standardised precipitation-evapotranspiration index. Available at: https://CRAN.R-project.org/package=SPEI

[B35] Shi J. Xiang W. Liu Q. Sher S. (2019). MtreeRing: An r package with graphical user interface for automatic measurement of tree ring widths using image processing techniques. Dendrochronologia 58, 125644. doi: 10.1016/j.dendro.2019.125644

[B36] Singh G. Mishra D. Singh K. Parmar R. (2013). Effects of rainwater harvesting on plant growth, soil water dynamics and herbaceous biomass during rehabilitation of degraded hills in rajasthan, India. For. Ecol. Manage. 310, 612–622. doi: 10.1016/j.foreco.2013.09.002

[B37] Thc T. Neefjes K. Hng T. T. T. Thng N. V. Hi C. H. (2015)Summary for policymakers on the special report on managing the risks of extreme events and disasters to advance climate change adaptation (SREX) (Accessed June 9, 2022).

[B38] Trugman A. T. Detto M. Bartlett M. K. Medvigy D. Anderegg W. R. L. Schwalm C. . (2018). Tree carbon allocation explains forest drought-kill and recovery patterns. Ecol. Lett. 21, 1552–1560. doi: 10.1111/ele.13136 30125446

[B39] Vicente-Serrano S. M. Begueria S. Lopez-Moreno J. I. (2010). A multiscalar drought index sensitive to global warming: The standardized precipitation evapotranspiration index. J. Clim. 23, 1696–1718. doi: 10.1175/2009JCLI2909.1

[B40] Wang H. Shao X. Jiang Y. Fang X. Wu S. (2013). The impacts of climate change on the radial growth of pinus koraiensis along elevations of changbai mountain in northeastern China. For. Ecol. Manage. 289, 333–340. doi: 10.1016/j.foreco.2012.10.023

[B41] Wang Y. Shao M. Sun H. Fu Z. Fan J. Hu W. . (2020). Response of deep soil drought to precipitation, land use and topography across a semiarid watershed. Agric. For. Meteorol. 282–283, 107866. doi: 10.1016/j.agrformet.2019.107866

[B42] Wang X. Yang B. (2021). Divergent tree radial growth at alpine coniferous forest ecotone and corresponding responses to climate change in northwestern China. Ecol. Indic. 121, 107052. doi: 10.1016/j.ecolind.2020.107052

[B43] Wang Z. Y. Yang B. Deslauriers A. Brauning A. (2015). Intra-annual stem radial increment response of qilian juniper to temperature and precipitation along an altitudinal gradient in northwestern China. Trees-Struct. Funct. 29, 25–34. doi: 10.1007/s00468-014-1037-7

[B44] Wang X. Yang B. Ljungqvist F. C. (2019). The vulnerability of qilian juniper to extreme drought events. Front. Plant Sci. 10. doi: 10.3389/fpls.2019.01191 PMC6777612 31611900

[B45] Wigley T. M. L. Briffa K. R. Jones P. D. (1984). On the average value of correlated time series, with applications in dendroclimatology and hydrometeorology. J. Appl. Meteorol. Climatol. 23, 201–213. doi: 10.1175/1520-0450(1984)023<0201:OTAVOC>2.0.CO;2

[B46] Yu J. Liu Q. (2020). Larix olgensis growth–climate response between lower and upper elevation limits: an intensive study along the eastern slope of the changbai mountains, northeastern China. J. For. Res. 31, 231–244. doi: 10.1007/s11676-018-0788-1

[B47] Yu D. Liu J. Benard J. L. Zhou L. Zhou W. Fang X. . (2013). Spatial variation and temporal instability in the climate–growth relationship of Korean pine in the changbai mountain region of northeast China. For. Ecol. Manage. 300, 96–105. doi: 10.1016/j.foreco.2012.06.032

[B48] Zeng X. Wei C. Liu X. Zhang L. (2020). Qinghai spruce (Picea crassifolia) and Chinese pine (Pinus tabuliformis) show high vulnerability and similar resilience to early-growing-season drought in the helan mountains, China. Ecol. Indic. 110, 105871. doi: 10.1016/j.ecolind.2019.105871

[B49] Zhao Y. Cai L. Jin Y. Li J. Cui D. Chen Z. (2021). Warming-drying climate intensifies the restriction of moisture on radial growth of pinus tabuli-formis plantation in semi-arid area of northeast China. Chin. J. Appl. Ecol. 32, 3459–3467. doi: 10.13287/j.1001-9332.202110.034 34676706

[B50] Zhirnova D. F. Belokopytova L. V. Barabantsova A. E. Babushkina E. A. Vaganov E. A. (2020). What prevails in climatic response of pinus sylvestris in-between its range limits in mountains: slope aspect or elevation? Int. J. Biometeorol. 64, 333–344. doi: 10.1007/s00484-019-01811-0 31691013

[B51] Zhou W. Chen N. (2018). Spatial distribution of extraterrestrial solar radiation and its spatial scale effect on rugged terrains. J. Geo-Inf. Sci. 20, 186–195. doi: 10.12082/dqxxkx.2018.170363

