# Supplementary material for: Microtopography mediates the climate–growth relationship and growth resilience to drought of Pinus tabulaeformis plantation in the hilly site
Source: Front Plant Sci. 2022 Nov 22;13:1060011. doi: 10.3389/fpls.2022.1060011 (PMC9723379; doi:10.3389/fpls.2022.1060011)
Supplement: Supplementary file 1 [file Image_1.pdf]

Supplementary Material

1 Supplementary Figures

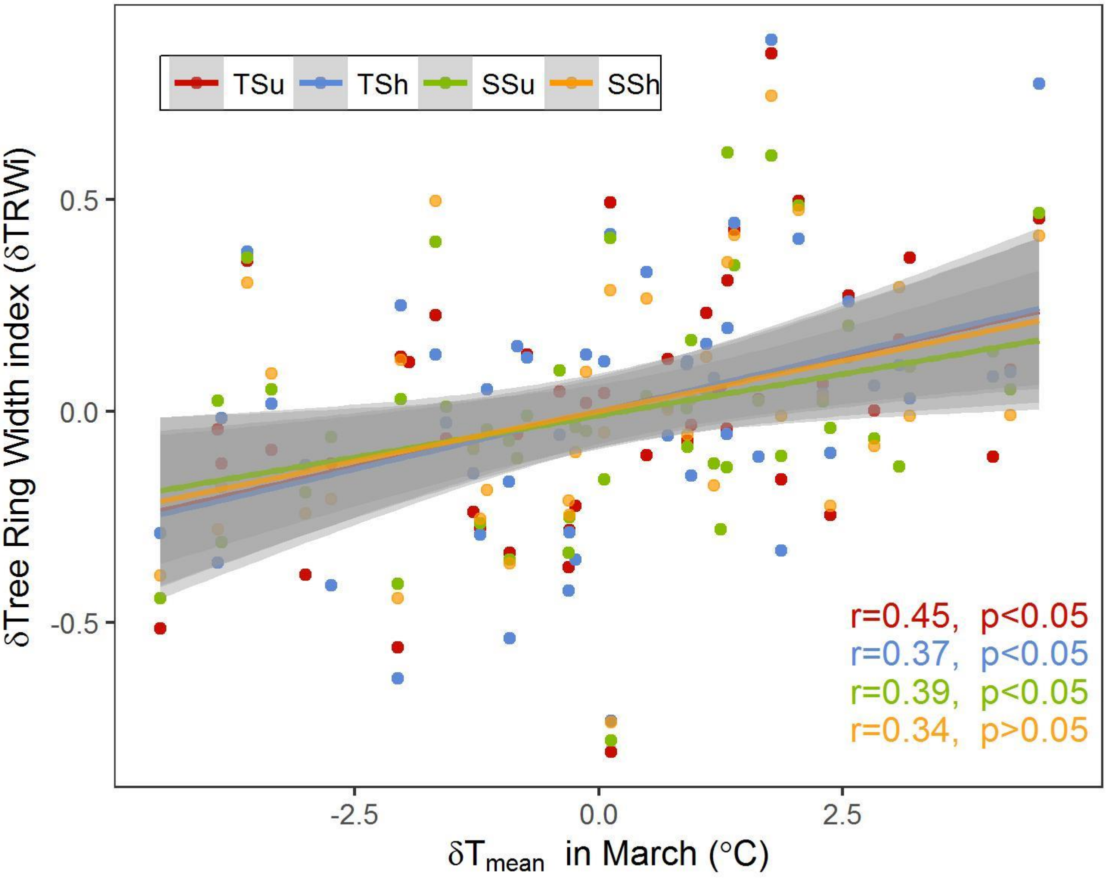

Supplementary Figure 1. Correlate the first-order difference of standard chronologies (STDs) of tree ring width index (TRWi) in all plots with that of monthly mean temperature (T_{mean}) in March.
